# Behavioral interventions for vaccination uptake: A systematic review and meta-analysis

**DOI:** 10.1016/j.healthpol.2023.104894

**Published:** 2023-09-04

**Authors:** Amyn A. Malik, Noureen Ahmed, Mehr Shafiq, Jad A. Elharake, Erin James, Kate Nyhan, Elliott Paintsil, Hannah Melchinger, Yale Behavioral Interventions Team, Fauzia A. Malik, Saad B. Omer

**Affiliations:** a Yale Institute for Global Health, New Haven, CT 06510, USA; b Analysis Group, Inc, Boston, MA 02199, USA; c UT Southwestern Peter O’Donnell Jr. School of Public Health, Dallas, TX 75390, USA; d Columbia University School of Public Health, New York, NY 10032, USA; e Columbia University Institute of Human Nutrition, New York, NY 10032, USA; f The Ohio State University College of Medicine, Columbus, OH 43210, USA; g Yale University, New Haven, CT 06510, USA

## Abstract

**Background:**

Human behavior and more specifically behavioral insight-based approaches to vaccine uptake have often been overlooked. While there have been a few narrative reviews indexed in Medline on behavioral interventions to increase vaccine uptake, to our knowledge, none have been systematic reviews and meta-analyses covering not just high but also low-and-middle income countries.

**Methods:**

We included 613 studies from the Medline database in our systematic review and meta-analysis categorizing different behavioral interventions in to 9 domains: education campaigns, on-site vaccination, incentives, free vaccination, institutional recommendation, provider recommendation, reminder and recall, message framing, and vaccine champion. Additionally, considering that there is variability in the acceptance of vaccines among different populations, we assessed studies from both high-income countries (HIC) and low- to middle-income countries (LMIC), separately.

**Findings:**

Our results show that behavioral interventions can considerably improve vaccine uptake in most settings. All domains that we examined improved vaccine uptake with the highest effect size associated with Provider Recommendation (OR: 3.4 (95%CI: 2.5-4.6); Domain: motivation) and Onsite vaccination (OR: 2.9 (95%CI: 2.3-3.7); Domain: practical issues). Although the number of studies from LMIC was smaller, the quality of studies was similar across HIC and LMIC. However, effect sizes were different.

**Interpretation:**

Our findings indicate that “provider recommendation” and “on-site vaccination” along with other behavioral interventions can be employed to increase vaccination rates globally.

## Introduction

Vaccines are one of the most effective and cost-effective tools available for preventing infectious diseases and save millions of lives each year.^1,2^ However, vaccine uptake is variable across different populations and vaccines. Globally, progress towards equitable vaccine coverage has been uneven.

There are multiple determinants of under-vaccination including inadequate supply of vaccines, and lack of awareness and education about vaccination. While some of the barriers to vaccine uptake are structural, others are related to human behavior.^3,4^ Behavioral science, which uses an interdisciplinary approach to systematically study human behavior, offers promise in designing interventions that use the behavioral and social determinants of vaccination to increase vaccine uptake. Behavioral science as a field has advanced significantly in the past decade, however, behavioral insights have been applied unevenly to immunization efforts.

While there have been a few narrative reviews on behavioral interventions to increase vaccine uptake, none have been systematic reviews and meta-analyses covering not just high but also low-and-middle income countries (LMICs).^5,6^

We present results from the first comprehensive systematic review and meta-analysis of the literature on behavioral insights-based interventions to increase vaccine uptake. This review aims to synthesize the evidence on which behavioral interventions work, and which could work to inform interventions at all levels of the vaccine delivery process and increase equitable vaccine coverage in LMICs. We assessed all articles published between 1990 and 2020 as described in methods and material. Our primary outcome of interest was the effects of behavioral interventions on vaccine uptake. We also considered articles discussing the effects of behavioral interventions on vaccine knowledge, attitude, and intent.

We classified interventions into nine domains: education campaigns, on-site vaccination, incentives, free vaccination, institutional recommendation, provider recommendation, reminder and recall, message framing, and vaccine champion. For studies that address multiple domains, we classified them under multiple domains.

## Methods

### Search strategy

We searched the database Medline on the platform Ovid, using the Medline All segment, which includes non-Medline PubMed records. While we considered searching additional bibliographic databases – in particular, Embase and PsycINFO – we decided that a comprehensive search strategy in a single database with robust subject indexing was sufficiently sensitive. Additional search on Embase did not add to the sensitivity of the search using the identified seminal studies (described below). Relevant material from journals not indexed in Medline (such as *Journal of Economic Behavior and Organization, Behavioral Science and Policy*, and *Health Communication*) was identified through expert knowledge and citation chaining.

We tested multiple preliminary searches. While the part of the search about vaccination was not complex to design, two methodological decision points emerged: how to operationalize the concept of behavioral approaches and whether to include a third concept (the outcome of vaccination uptake, a broader set of outcomes including vaccine knowledge, attitudes, and practices as well as uptake, or a study design filter for intervention studies).

To test different iterations of the search strategy, we identified 43 seminal studies on behavioral interventions for vaccine uptake. Of these, 39 were available in Medline, and we used these to evaluate the sensitivity of different iterations of the search strategy. We also noted the screening workload of different approaches. Some of the approaches we considered are presented in the table below. Some of the approaches we considered are presented in Supplemental Table 1 with complete details in Appendix A of the Supplement. Operationalization III performed the best out of these approaches and was the basis for the final version of the search.

Ultimately, we added restored two of the behavior terms from Operationalization II to Operationalization III. With respect to the final validation set of 39 records available in Medline, this approach to the behavioral concepts retrieved 35 of them, or 90%. The whole search strategy retrieved 33 (85%) of the validation set. Its three concepts (vaccines, intervention studies, and behavioral approaches) are operationalized with keywords and controlled vocabulary terms.

Documents dated prior to 1990 and documents that solely focused on animals without considering humans were not included in the search results. On March 12, 2020, the search yielded a total of 14,753 records, out of which 47 duplicates were identified and removed. These records were then uploaded to Covidence for further examination. Along with the database search, 412 additional studies with potential relevance were discovered through expert knowledge and citation chaining, and they were also uploaded to Covidence for screening.^7^

The final search is presented in Appendix B. The search can be rerun by copying the column of queries into https://tools.ovid.com/ovidtools/launcher.html. This search history can be translated into PubMed format as follows:

(vaccines[mh] OR immunization[mh] OR vaccin*[tw] or immunis*[tw] or immuniz*[tw] or inoculat*[tw]) AND (intervention[tw] or interventions[tw] or treatment[tw] or treatments[tw] or group[tw] or groups[tw] or trial[tw] or trials[tw] or program[tw] or programs[tw] or programme[tw] or programmes[tw] or evaluat*[tiab] or experiment*[tiab]) AND (behavior[mh] OR behav*[tw] OR incentiv*[tw] OR psychology[subheading] OR motivat*[tw] OR motivation[mh]) NOT (animals[mh:noexp] NOT humans[mh]) AND (1990:3000[pdat])

### Study selection

We considered articles focused on a behavioral insights intervention related to vaccination. Behavioral insights utilize principles from psychology and economics to understand and influence human behavior, informing the design of interventions and policies that align with how people behave. We excluded articles that were explanatory or not based on interventions, as well as those that didn’t examine vaccine uptake, intent, knowledge, or attitudes as outcome measures, or were not in English. We also did not include systematic reviews and meta-analyses. Two reviewers independently evaluated the titles and abstracts of the selected articles for manual screening to determine the final studies to be included.

### Study Outcomes of interest

In this review, the main focus was on vaccine uptake as the primary outcome of interest, while vaccine knowledge, attitudes, and intent were considered as secondary outcomes of interest. Each study had its own criteria for defining the outcome. Some studies categorized outcomes based on self-reports, while others relied on clinical or insurance records.^7^

### Data extraction

Two coders independently reviewed the full text of all studies and extracted data using a Google Form specifically designed for the data extraction. Any disagreements were resolved by reviewing the study with a third judge and a decision was mutually agreed upon. We coded the study characteristics including population, age, study type, inclusion of a control group, vaccine type, country of the study, setting (rural vs urban), outcomes assessed, and strength of the study using GRADE criteria (Appendix C). Each individual study was assessed based on the study design, strengths and limitations and was graded. If there were more than 8 high GRADE studies in any domain, the overall GRADE score for the domain was assigned high.

#### Interventions

We categorized interventions into different domains, including education campaigns (providing education on vaccination, disease, and how vaccine work), on-site vaccination (providing vaccine at workplace or places of worship), incentives (offering financial incentives for vaccination), free vaccination (providing vaccination free of cost), institutional recommendation (recommendation made by the institution that person works at especially for healthcare providers), provider recommendation (recommendation by doctor/nurse), reminder and recall (reminders for vaccination), message framing (persuasion messaging, gain vs loss framing of the vaccine), and vaccine champion (institutional or self-appointed champion that encouraged vaccination), based on their characteristics.^7^ These domains emerged thematically during qualitative review of the studies included in the systematic review. Studies addressing multiple domains were classified under each relevant domain.

The domains that we used map on the World Health Organization’s Behavioral and Social Drivers of Vaccination (BeSD) model^8^ as follows: What people think and feel (educational campaigns), social processes (provider recommendation), motivation (vaccine champion), and practical issues (on-site vaccination, free vaccination). This provides for alignment with current work and will provide options for different levers to pull.

### Data synthesis

In our primary analyses, we conducted a meta-analysis for each intervention domain using a random effect model with the outcome of interest being vaccine uptake. We used the inverse variance method to generate pooled odds ratios (ORs) with 95% confidence intervals.

We extracted odds ratios or raw numbers from individual studies to calculate a summary odds ratio for each intervention’s effect on vaccine uptake (self-reported or from clinical or insurance records). Outcome was defined as per each study’s criteria. Studies that did not provide enough information for calculation of odds ratios were excluded from this meta-analysis.

For sensitivity analysis, we removed the outlier studies defined as a study whose 95% confidence interval does not overlap with the confidence interval of the pooled effect and recalculated the summary measures. We assessed the presence of publication bias using funnel plot asymmetry and Egger’s regression. For domains with indication of publication bias, we used the trim and fill method to calculate adjusted ORs and 95% CIs. All analyses were conducted in R (version R.4.1.2.) using the packages “meta”^9^ and “metasens”.^10^

The study was funded by Bill & Melinda Gates Foundation. Funder played no part in study design, data collection, analysis, interpretation, and manuscript preparation.

## Results

**Figure 1.**
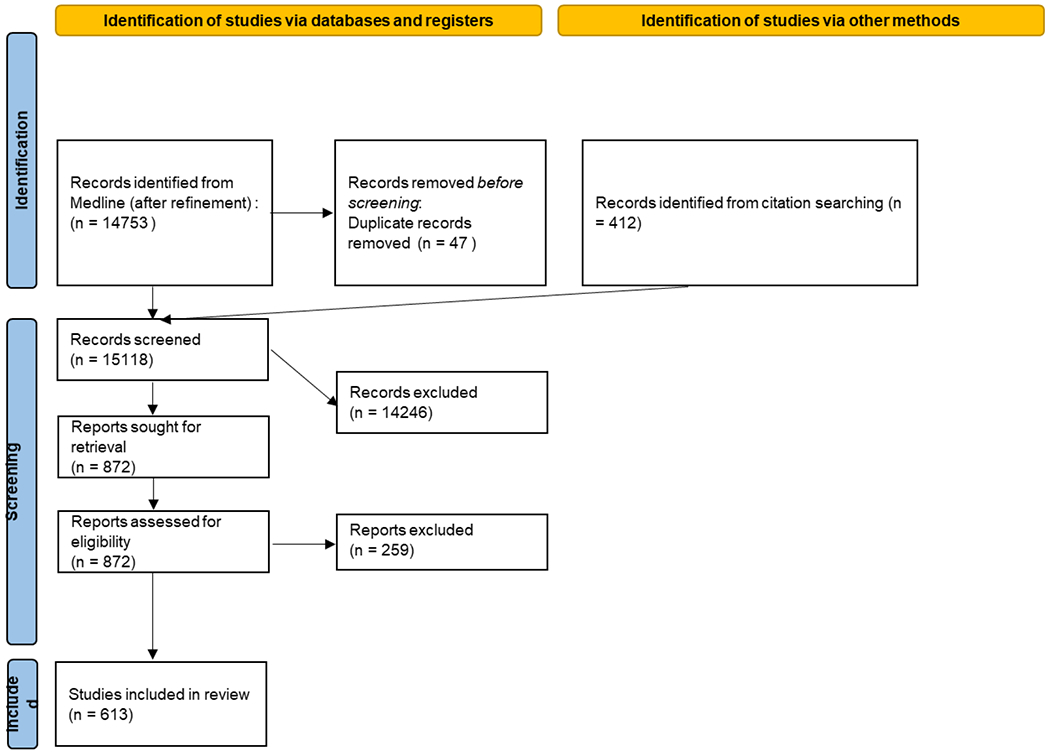
Studies included and excluded at each stage of review process.

After search criteria refinement, we identified 15,118 studies of which 872 were selected for full text review after title and abstract screening. Of these 872 studies, 613 studies met the inclusion criteria and were included in this review (Figure 1). These 613 represent 64 countries globally with 37 countries being classified as Higher Income (HIC) and 27 as Low and Middle Income (LMIC) countries as per the World Bank Criteria (Supplemental Figure 1). Of these 613 studies, 474 (77%) reported on vaccine uptake,^11–485^ 145 (24%) on vaccine attitude,^12, 14, 20, 25, 26, 39, 46, 48, 81, 107,113,124,145,150,166–170,172,174,175,178–180, 184,186,187,191,195,202,216,236,262,269,273,287,288,292,293,297,300,302,304,310,317,318,320,322,323,328,329,335,341,345,346,352,353,359,363–365,368,371,383,404,426,428,435,444,450,486–559^ 139 (23%) on vaccine knowledge, ^13,14,20,25,26,28,31,39,40,48,55,70,113,124,131,134,142,143,145,150,151,166–169,172,175,178,180,183,186,187,189,191,195,199,202,216,218,247,256,266,276,280,282,283,288,295,300,302,304,308,311,312,322,327,343,345,346,352,356,360,386,396,403,404,425,442,486–489,492,493,498,499,501,503–507,509,510,514–516,518–521,523,526–528,531–533,535,536,541,545–547,549,550,552,556,557,560–591^ and 122 (20%) on vaccine intent.^12,13,20,36,44,48,56,64,67,75,77,81,111–113,115,116,124,131,145,151,167,175,178,184,186,187,191,192,196,205,218,220,236,247,311,312,320,322,326,327,329,334,346,353,365,368,384,435,464,470,471,474,476,486,498,500–502,505,512–514,517–520,523,525,530–533,537,539–541,546,551,553,554,560,562,563,565,569,572,578,588,590,592–622^ Studies focused both on providers as well as patients and included different target populations: Children (n=304, 49.6%), healthcare workers (n=70, 11.4%), general adults (n=200, 32.6%), adults over 65 years of age (n=57, 9.3%), and adults with pre-existing conditions (n=47, 7.7%).

Most of the studies were classified as educational campaigns (n=315, 51%) ^13,14,17,19,20,22,25,26,31–34,36,39,40,43,47,48,50,55,58–62,66,69,70,72–75,79–85,90–93,96,100–103,105,106,112,115,116,120,121,123–125,128,131,133,134,141–146,148,150–154,156,160,164–169,171,172,175,178–180,182–187,189,190,196,199,201–205,207,212,215,216,219,220,222–224,231–237,240,241,245–249,252,253,256–260,262,265,267,269–274,276–278,282,283,285,288,290–293,295,297–300,304,305,307,308,310–312,316,317,320,322,327,334–336,338,344–347,352,355–358,360,362,363,365,366,369,370,373,377,379–383,385,386,388,389,391,399,401–406,410,420,423,425,428,429,433,436,437,442,445–447,453,462,470–477,482,484,486,488,489,492,493,496,497,499,501,503–507,509–511,520,524–529,531,533–536,539–541,545–550,552,557–559,561–580,583,584,586–593,595,597,602–605,614,616,619,623^ followed by reminder and recall (n=155, 25%) ^15,18,21,26,28–33,35,40,47,50,54,56,59,64,67,70,72,77,86,97–99,105,108,114–116,123,129,132,133,135,137–139,141,143,146–148,153,160,166,174,178,179,181,188,193,194,202,206,209,210,221,225,229,232,233,238,248,253,262–264,268,271,272,276,278,279,285–290,294–297,302,303,305,307,309,310,313,314,317,319,320,326,329–331,335,337,342,343,345,348,351,360,364,366,372–379,381,384,385,389,393,394,397–399,403–405,408,409,412,416,419,431,434,436,438,440,441,449,457,458,463,465,468,473,480,515,537,539,549,599^, incentives (n=102, 17%) ^11,12,16,22–24,27,29,39,43,44,65,70,78,83,91,103,104,106,109,114,115,122,125–127,130,132,136,140,141,147,149,150,152,155,157,158,161,163,173,174,190,195,197,211,214,218,220,226,228,239,254,264,265,268,280,284,289–292,294,298,302,303,305,306,329–333,335,339,341,349–352,362,371,372,396,400,406,414,415,417,424,427,436,437,439,448,452,460,464,516,542,543,598^, message framing (n=98, 16%) ^20,37,40,45,46,53,57,61,74,81,83,88,94,110,111,118,150,151,167,179,185,188,196,197,202,220,222,229,241,256,283,292,306,318,322,343,353,357,359,390,421,422,425,447,451,471,484,491,494,495,498,500,502,504,509,512–514,517,518,529,530,532,538,541,542,544,545,551,553–556,559,569,586,594–596,598,600,601,605–613,615,617,618,620–622^ on-site vaccination (n=71, 12%) ^20,26,33,38,39,43,49,56,72,89,94,101,113,119,121,143,150,162,176,179,190,191,208,209,217,223,224,234,244,250,254,255,264–266,269,289,290,292,305,306,324,336,338,344,347,357,360–362,364,369,383,387,388,392,395,406,427,436,450,453–455,461,469,474,475,479,483,490,560,581,597^, free vaccination (n=63, 10%) ^20,21,28,41,48,49,54,72,100,123,150,157,165,168,170,190,191,198,207,209,219,225,240,269,291–293,306,307,314,317,318,323,336,338,341,364,366,383,387,388,392,404,417,422,430,432,436,441,443,447,448,458,459,461,462,469,477,479,490,555,599,605^, provider recommendation (n=55, 9%)^19,21,51–53,56,59,61,67,68,71,72,74,87,103,135,147,150,154,207,213,230,233,242,258,259,272,277,279,281,310,325,328,340,358,366,368,372,382,387,407,411,418,422,424,427,440,444,470,519,586,597,599,614,616^, institutional recommendation (n=49, 8%) ^22,42,43,72,95,150,154,177,190,203,243,246,257,259–261,265,267,270,271,274,275,277,278,286,291,296,304–306,315,321,328,335,342,354,355,363,367,411,412,426,435,448,456,460,468,487,604, and vaccine champion (n=23, 4%; table 1)26,84,106,113,150,223,225,227,250,253,258,269,292,301,369,379,467,474,475,522,524,547,597,598^. Below, we describe the domains in detail and highlight some studies across different countries as examples of how different techniques have been used to increase vaccine uptake.

### Educational Campaigns

Educational Campaigns was the most represented domain in our review. Studies in this domain came from 53 countries with 20 countries classified as LMIC (Table 1). Most (n=273, 87%) of the studies were from HIC. Techniques used in this domain included informational sessions/classes, lectures, workshops, informational leaflets/handouts, and online training.

In a cluster randomized trial in Pakistan, Andersson et al implemented three structured discussions highlight vaccine uptake from a baseline survey they conducted, cost and benefits of childhood vaccination and local action plans with males and females separately in the intervention clusters. The intervention led to a 2-to-3-fold increase in measles and diphtheria-pertussis-tetanus (DPT) vaccination.^186^

In a school-based cluster randomized trial in Sweden, Grandhal et al used school nurses to deliver a 30 min face to face information session on HPV, including cancer risks and HPV prevention. After the intervention, the proportion of vaccinated girls in the intervention group increased by 6.5 percentage points in the intervention group while there was no change in the control group.^391^

**Figure 2.**
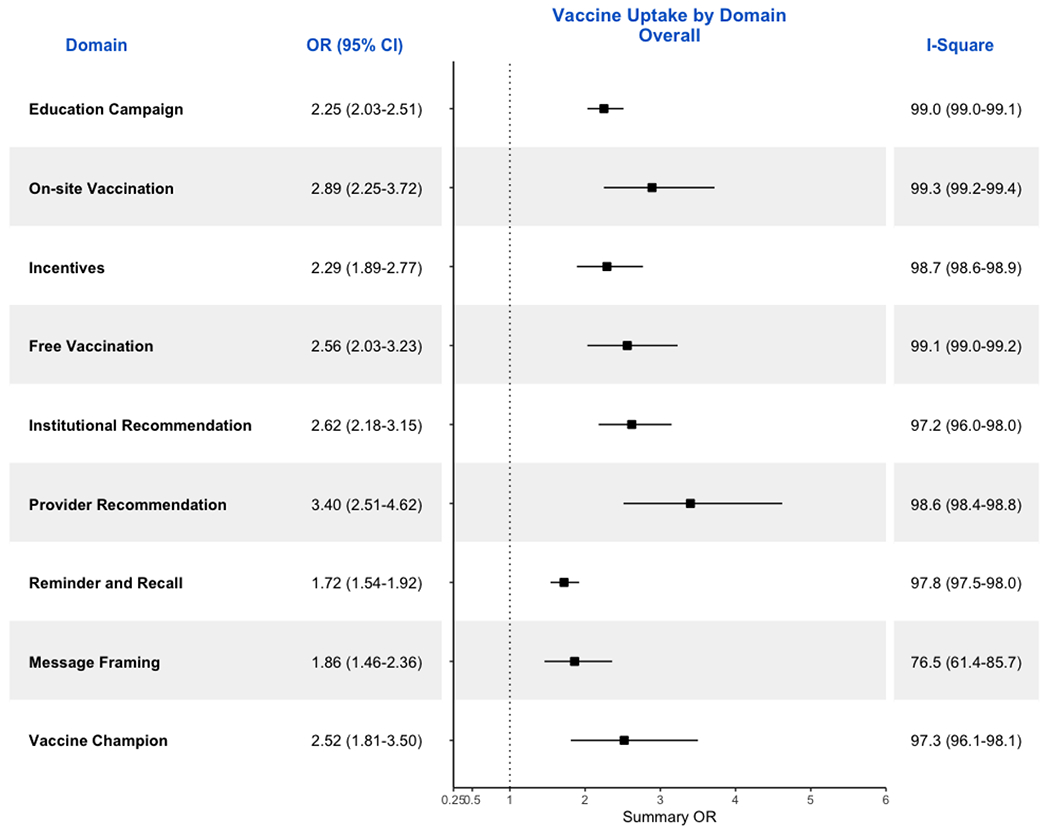
Forrest Plot showing ORs and respective 95% CIs for all studies included in the meta-analysis by domains

**Figure 3.**
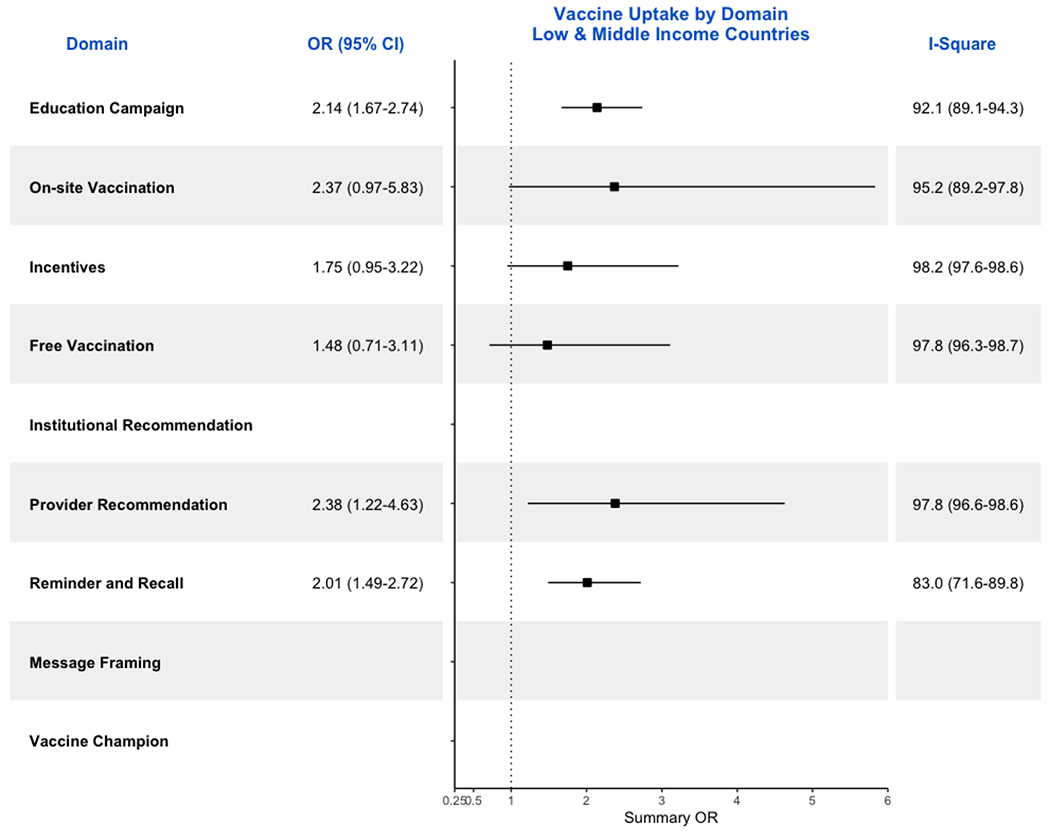
Forrest Plot showing ORs and respective 95% CIs for all studies from LMICs included in the meta-analysis by domains

**Figure 4.**
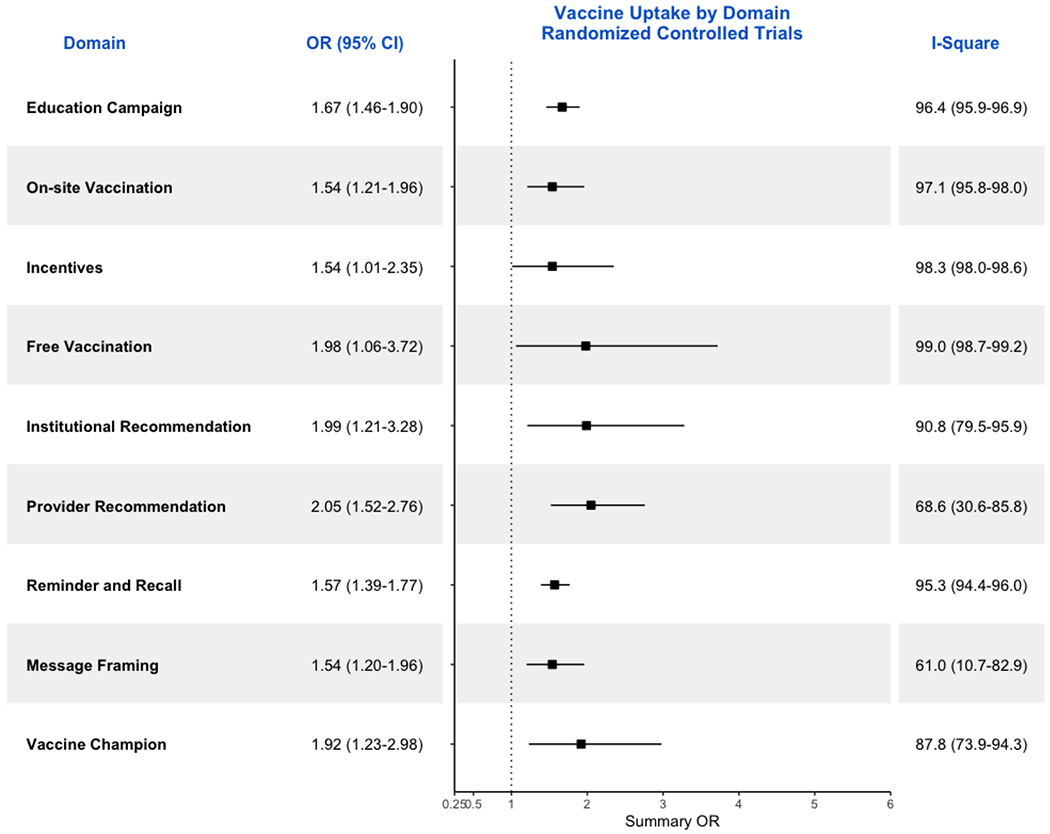
Forrest Plot showing ORs and respective 95% CIs for all RCTs included in the meta-analysis by domains

The overall GRADE score for this domain was high. There were 147 studies with enough information to calculate an OR. The overall pooled OR was 2.3 (95%CI: 2.0-2.5; Figure 2). Just considering studies in LMIC, the pooled OR was 2.1 (95%CI: 1.7-2.7; Figure 3). Restricting to RCTs, the pooled OR was 1.7 (95%CI: 1.5-1.9; Figure 4). However, there was huge heterogeneity in the studies as shown by the I^2^ across all strata.

### Reminder and Recall

Reminder and recall include all studies that provided a reminder for vaccination. Studies in this domain represented 24 countries with 9 being LMIC (Table 1). Techniques used for reminder and recall included text message reminders, telephone calls, letters, reminder services/apps and appointment cards.

Gibson et al randomized villages in Kenya to 4 groups: control, short message service (SMS) reminder only, SMS plus a 75 Kenya Shilling (KES) incentive, and SMS plus 200 KES. The study found that children randomized to SMS plus 200 KES group were 1.09 times more likely to be completely immunized as compared to the control group (RR: 1.09; 95%CI: 1.02 – 1.16).^330^

In a randomized controlled trial in Pakistan, mother-child pairs were randomized to 1 of the 4 groups: randomized to four study groups: redesigned card, center-based education, combined intervention, and standard care. Children in the redesigned card (RR: 1.7; 95%CI: 1.5-2.0), educational (RR: 1.5; 95%CI: 1.3-1.8) and combined intervention (RR: 1.7; 95%CI:1.4-2.0) were more likely to complete the third dose of DTP vaccine (DTP3) than children in the standard of care arm.^377^

The overall GRADE score for this domain was high. There were 91 studies with enough information to calculate an OR. The overall pooled OR was 1.7 (95%CI: 1.5-1.9; Figure 2). Just considering studies in LMIC, the pooled OR was 2.0 (95%CI: 1.5-2.7; Figure 3). Restricting to RCTs, the pooled OR was 1.6 (95%CI: 1.4-1.8; Figure 4). However, there was huge heterogeneity in the studies as shown by the I^2^ across all strata.

### Incentives

Incentive studies represented 26 countries with 14 being LMIC (Table 1). Incentive types included financial incentives, food vouchers/packages, transportation, and in-kind incentives.

A study from rural Nigeria randomized women to receive cash incentives if they completed their tetanus toxoid vaccination into 3 groups: 5 Nigerian naira (C5), 300 naira (C300), and 800 naira (C800). Investigators found that women in C300 and C800 groups were more likely to receive the vaccine as compared to women in the C5 group. The study also noted that transportation costs to be a significant barrier preventing women from receiving vaccines at clinic and the cash incentive compensated for the transportation cost.^125^

A randomized controlled trial in 2 inner-city health services in Sydney, Australia randomized Serologically confirmed HBV-susceptible people who inject drugs to receive 30 Australian Dollars cash following receipt of vaccine doses two and three of HBV vaccine or standard care. The completion rate for all 3 doses in the intervention arm was 87% compared to 66% in the control arm.^27^

The overall GRADE score for this domain was high. There were 51 studies with enough information to calculate an OR. The overall pooled OR was 2.3 (95%CI: 1.9-2.8; Figure 2). Just considering studies in LMIC, the pooled OR was 1.8 (95%CI: 1.0-3.2; Figure 3). Restricting to RCTs, the pooled OR was 1.5 (95%CI: 1.1-2.4; Figure 4). However, there was huge heterogeneity in the studies as shown by the I^2^ across all strata.

### Message Framing

Studies in this domain represented 27 countries with 3 being LMIC (Table 1). Modalities used included persuasive messages, gain vs loss framed messages, assertive messages, and self-affirmation.

Chien used a 2 by 3 factorial design to assess the impact of gain vs loss framed messages and color configuration in online banners on persuasion for flu vaccination. The study found that loss framed messages using white text on red background improved persuasion compared to gain framed messages.^538^

A randomized controlled trial in India provided mothers face-to-face information on the benefits of tetanus vaccine. The trial had 3 arms: mothers in the first arm received information framed as a gain (e.g., the child is less likely to get tetanus and more likely to be healthy if vaccinated), mothers in the second arm received information framed in terms of a loss (e.g., the child is more likely to get tetanus and suffer ill health if not vaccinated), and the third arm was the control group. The pooled RR for the 2 intervention groups was 1.7 (95%CI: 1.3-2.3) for full immunization. There was no difference between the 2 intervention arms.^118^

The overall GRADE score for this domain was high. There were 15 studies with enough information to calculate an OR. The overall pooled OR was 1.9 (95%CI: 1.5-2.4; Figure 2). As there was only 1 study with information to calculate an OR from LMIC, LMIC specific pooled OR was not calculated. Restricting to RCTs, the pooled OR was 1.5 (95%CI: 1.2-2.0; Figure 4). However, there was huge heterogeneity in the studies as shown by the I^2^ across all strata.

Gain vs loss framed messages did not differ in improving vaccine uptake.

### On-site Vaccination

The 71 studies in this domain came from 25 countries of which 11 were LMIC (Table 1). Vaccination sites included churches/places of worship, schools, workplaces, hospitals/local clinics and community centers.

Daniels et al provided on-site vaccination at faith-based institutions (churches) in African American and Latinx communities in a cluster randomized controlled trial in California, USA. The study found a difference of between 31 and 34 percentage points for pneumococcal and influenza vaccination uptake in intervention vs control groups.^143^

A teaching hospital in Nigeria implemented a vaccine program where free vaccinations were provided on-site to the hospital staff. The level of participation among healthcare workers was high, with 91.9% of the target group receiving at least one dose of the Hepatitis B vaccine.^490^

The overall GRADE score for this domain was high. There were 23 studies with enough information to calculate an OR. The overall pooled OR was 2.9 (95%CI: 2.3-3.7; Figure 2). Just considering studies in LMIC, the pooled OR was 2.4 (95%CI: 1.0-5.8; Figure 3). Restricting to RCTs, the pooled OR was 1.5 (95%CI: 1.2-2.0; Figure 4). However, there was huge heterogeneity in the studies as shown by the I^2^ across all strata.

### Free Vaccination

Studies in this domain came from 22 countries of which 5 were LMIC (Table 1).

In Colombia, Bogota Health Department collaborated with a foundation to provide a free multi-dose vaccination program for Hepatitis B among the female and transexual sex workers. The study found a higher adherence rate (37.7%) for the vaccination program among these marginalized communities compared to other programs.^198^

In a cluster randomized controlled trial in France, Launay et al evaluated the impact of free on-site Hepatitis B vaccinations, training on epidemiology and risk-factors or both for healthcare workers on HBV vaccination acceptability in high-risk adults consulting in 12 free and anonymous HIV and hepatitis B/C testing centres. The study showed that training and free on-site vaccine availability was more effective than free on-site vaccine availability alone (RR: 1.14; 95%CI: 1.02-1.26) to increase vaccination acceptability resulting in a 29.8 percentage point increase in vaccine coverage. There was no effect from healthcare worker training alone.^240^

The overall GRADE score for this domain was high. There were 33 studies with enough information to calculate an OR. The overall pooled OR was 2.6 (95%CI: 2.0-3.2; Figure 2). Just considering studies in LMIC, the pooled OR was 1.5 (95%CI: 0.7-3.1; Figure 3). Restricting to RCTs, the pooled OR was 2.0 (95%CI: 1.1-3.7; Figure 4). However, there was huge heterogeneity in the studies as shown by the I^2^ across all strata.

### Provider Recommendation

The 55 studies in this domain came from 14 countries of which 3 were LMIC (India, Nigeria, and Sudan) (Table 1). Providers included family practitioners, nurse practitioners, community clinic staff, immunization nurses and pharmacists, while techniques included standing orders.

Ansari et al report on a study conducted in India where an intervention was implemented to promote polio vaccination among resistant families. A team of healthcare workers conducted house to house visits identifying families who refused to give polio drops to the children. These families were again visited by medical interns, who provided health education and vaccine promotion/recommendation. Families still resistant to vaccination were visited once more by another team. Of the resistant families, 79.3% were persuaded to receive the polio vaccinations after two rounds of visits.^382^

Three hundred and forty-six unvaccinated patients with inflammatory bowel disease (IBD) were randomized to either routine clinical care or intervention group receiving additional education by a nurse with help of an information brochure and vaccination card in Belgium. Eight months later, 33% of intervention group versus 6% of control group had followed vaccination recommendations.^154^

The overall GRADE score for this domain was high. There were 33 studies with enough information to calculate an OR. The overall pooled OR was 3.4 (95%CI: 2.5-4.6; Figure 2). Just considering studies in LMIC, the pooled OR was 2.4 (95%CI: 1.2-4.6; Figure 3). Restricting to RCTs, the pooled OR was 2.1 (95%CI: 1.5-2.8; Figure 4). However, there was huge heterogeneity in the studies as shown by the I^2^ across all strata.

### Institutional Recommendation

Of the 49 studies in the domain, 43 (88%) were from HIC countries representing 10 countries. (Table 1). Techniques used included mandatory declination, vaccine mandates, supervisory reminders, and visual cues.

Belcher report on a randomized controlled trial in Seattle with 3 interventions: 1) a physician-oriented education and motivation model with periodic feedback about performance, 2) a patient education model with mailed informative brochure, and 3) a health promotion clinic. Only the use of the health promotion clinic model was found to increase prevention rates (including vaccination rates) three-folds and had a sustained impact over five years.^246^

In San Antonio, Texas, a study done on healthcare workers to improve influenza vaccination rates intervened using support of leadership, distribution of vaccine kits, grand rounds presentations and email and phone reminders. The study found a significant increase in vaccination rates among the staff going from 58.8% to 76.6%.^259^

The overall GRADE score for this domain was high. There were 10 studies with enough information to calculate an OR. The overall pooled OR was 2.6 (95%CI: 2.2-3.2; Figure 2). As there was only 1 study with information to calculate an OR from LMIC, LMIC specific pooled OR was not calculated. Restricting to RCTs, the pooled OR was 2.0 (95%CI: 1.2-3.3; Figure 4). However, there was huge heterogeneity in the studies as shown by the I^2^ across all strata.

### Vaccine Champion

There were 6 countries represented in this domain with Nigeria being the only LMIC. Twenty-one (91%) of the studies were from HIC (Table 1). Champions included nurses, peers, community leaders, religious leaders and celebrities.

Slaunwhite et al report on a study from Canada where health workers were recruited to be champions to motivate members of their work units to get vaccinated against influenza. These champions were provided a brief training session to increase awareness. The results showed a significant increase in vaccine rates among the units with vaccine champions.^301^

In a randomized controlled trial in Georgia, USA, expecting mothers were randomized to an intervention bracket including identification of a vaccine champion, provider-to-patient talking points, educational brochures, posters, lapel buttons, and iPads loaded with a patient-centered tutorial. The rates of influenza and Tetanus vaccines were higher among the intervention groups as compared to the control group.^258^

The overall GRADE score for this domain was high. There were 9 studies with enough information to calculate an OR. The overall pooled OR was 2.5 (95%CI: 1.8-3.5; Figure 2). As there was only 1 study with information to calculate an OR from LMIC, LMIC specific pooled OR was not calculated. Restricting to RCTs, the pooled OR was 1.9 (95%CI: 1.2-3.0; Figure 4). However, there was huge heterogeneity in the studies as shown by the I^2^ across all strata.

### Interventions with limited evidence of effectiveness

Our review shows that incentives need to be valued by participants for them to be effective. In affluent or high-income communities, incentives did not show a significant increase in vaccine uptake amongst healthcare workers.

Decisional aids and self-assessments amongst Educational Campaigns were shown to be less effective in improving vaccine uptake. Educational messages also need to be paired with self-efficacy or response-efficacy messages as on their own, educational messages did not improve vaccine uptake.

Studies did not evaluate vaccine uptake in quality improvement programs generally. Limited available evidence did not show quality improvement programs increase vaccine uptake.

### Sensitivity Analysis

**Figure 5.**
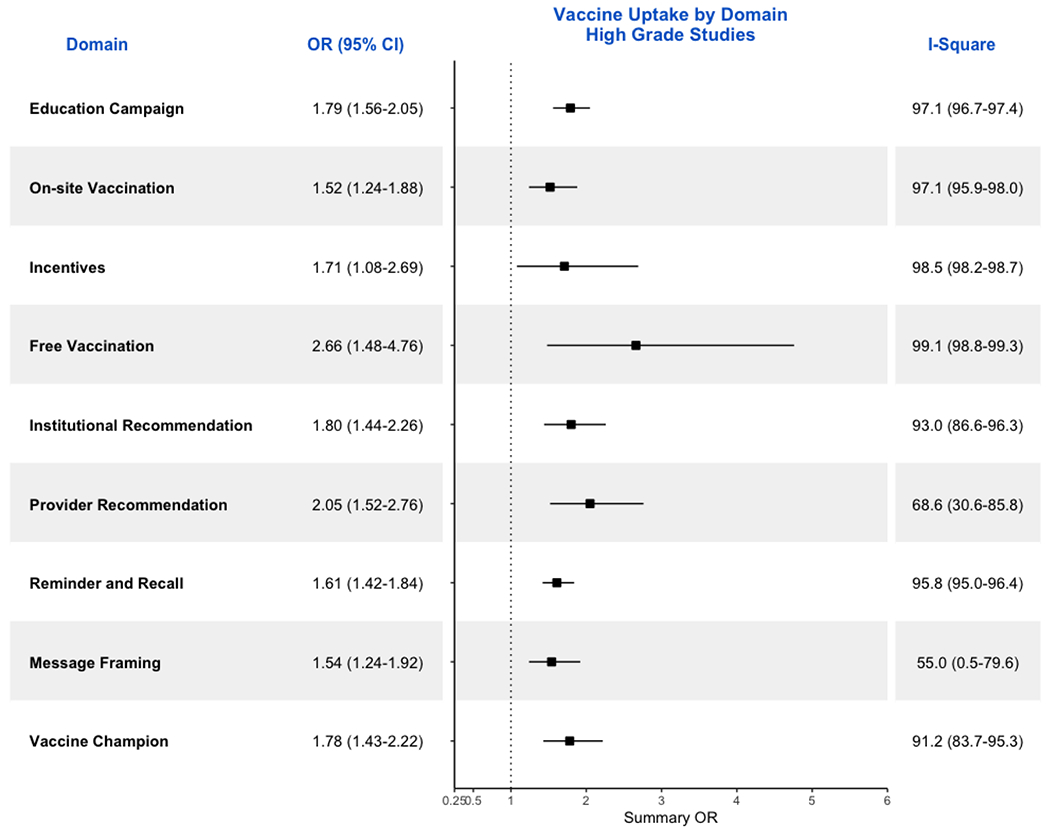
Forrest Plot showing ORs and respective 95% CIs for all high-GRADE studies included in the meta-analysis by domains

In sensitivity analysis, we recalculated the ORs and 95% CIs (Figure 5) for all studies in domains that were Graded high. There was no difference in ORs for RCTs and high-GRADE studies (Figures 4 and 5). This is to be expected as one of the grading criteria for a study to be Graded high is RCT.

Supplement Figure 2 shows the ORs and 95% CI for RCTs conducted in LMICs. The results are comparable to when restricting to studies conducted in LMICs (Figures 3 and 6).

### Sensitivity Analysis and Publication Bias

In sensitivity analysis, we removed the outlier studies in each domain and recalculated the pooled ORs and 95%CI (Supplement Table 2). The ORs and 95%CI were very similar to those calculated from the full study set however the I^2^ improved for all domains.

We assessed the presence of publication bias using visual representation of the studies (funnel plots; Supplement Figure 3) and Egger’s test (Supplement Table 3). Based on these analyses, we suspected presence of publication bias for 4 domains. We used trim and fill with outliers removed to recalculate the ORs and 95%CI to adjust for the possibility of publication bias. The adjusted pooled ORs were Education 2.1 (95%CI:2.0-2.2), Provider Recommendation 3.4 (95%CI:2.8-4.1), Institutional Recommendation 2.4 (95%CI:1.8-3.1), and Vaccine Champion 2.0 (95%CI:1.4-2.9). There was no indication of publication bias when we restricted our analysis to just RCTs (Supplement Table 3).

## Discussion

Our results show that behavioral interventions can improve vaccine uptake considerably. All domains that we examined improved vaccine uptake with the highest effect size associated with Provider Recommendation (OR: 3.4; Domain: motivation) and Onsite vaccination (OR: 2.9; Domain: practical issues).

Healthcare workers are the most trusted source of information for health-related knowledge including vaccination as shown in multiple studies. Hence, if a healthcare provider recommends an action such as vaccination, individuals are more likely to adopt it increasing vaccine uptake. A previous systematic review and meta-analysis also demonstrated a strong and consistent association between provider communication and HPV vaccination initiation, completion, and follow-through.^624^ Standing orders that authorize health care personnel to assess a patient’s immunization status and administer vaccinations according to a pre-approved protocol have shown improvement in vaccine uptake both individually and when linked to reminder and recall. The evidence of their effectiveness is based on mostly programmatic studies/retrospective reviews.

Onsite vaccination provides easy access to vaccines near the patient, eliminating extra effort on their part, travel time and cost, leading to increased vaccine uptake. Onsite vaccination can also be combined with free vaccination further reducing the cost for the patient.

We found that educational interventions specifically designed to cater to specific target populations were effective in improving vaccine knowledge associated with improved vaccine uptake. This indicates a need for specifically designed campaigns for selected populations after identifying their needs to improve vaccine uptake.

When considering reminder and recall, both patients and provider reminder and recall systems are important. Reminder and recall interventions combined with other interventions such as incentives and education are more likely to increase vaccine uptake and should be offered as a package of interventions. Similarly, financial incentives both at the level of the provider and at the level of the consumer can improve vaccine uptake. There also seems to be a dose-response relationship between the amount of incentive and vaccine uptake. Each implementing region will need to consider the optimum number of financial incentives for their settings.

Message framing interventions can improve vaccine knowledge and acceptance depending on the other variables in the study and use of appropriately framed messages for target populations. However, some types of message framing can have negative effects on vaccine acceptance. Hence, such interventions need to be considered carefully before deployment and can be combined into a package of interventions combining other domains together.

Few of our included studies focused on message framing, institutional recommendation, and vaccine champions from LMICs that look at vaccine uptake as an outcome. The evidence from HICs for these domains is strong for vaccine uptake but further studies need to be conducted from LMIC to evaluate these interventions in that context. The quality of studies from HICs and LMICs was generally similar. However, the overall number of studies from HIC (n=523) was almost 6 times higher and many LMICs were not represented. This points to a need to conduct and publish more studies in LMIC settings as context may influence both behavioral interventions and their association with vaccine uptake.

Strengths of our study include a comprehensive review of the behavioral interventions focused on vaccine uptake. To our knowledge, this is the first systematic review and meta-analysis on the subject. Our search strategy was highly sensitive and inclusive, and we are confident that we captured a majority of the studies published on the topic in our review. We assessed the quality of the studies using a tool that is widely used for assessing quality of evidence and making recommendations and should be readily interpretable by different stakeholders including the policymakers. All domains had multiple high-quality studies resulting in a GRADE of high for each domain.

Limitations of our work include that not all studies provided enough information for calculating a summary measure for vaccine uptake and hence were not included in the meta-analysis. These studies were included in the systematic review. There was considerable heterogeneity in each domain. This is expected as the interventions considered have been used for different populations in many different settings and have been examined using a variety of study designs. Additionally, while our study aimed to provide a comprehensive review, there is potential heterogeneity and publication bias from high-income countries which could impact the generalizability of the findings, particularly in LMICs. We provide effect estimates by different settings and study design as sensitivity analyses. Although we searched a single database for studies, we used a comprehensive search strategy with robust subject indexing that was sufficiently sensitive in capturing the relevant studies. Further, to mitigate this, we assessed publication bias and corrected for it using a scientifically robust method (Trim and Fill) and present the corrected ORs for domains with publication bias indicated.

## Conclusions/Policy Recommendations

Our results show that behavioral interventions can be used to increase vaccine uptake in most settings, particularly provider recommendations and on-site vaccination. With on-site vaccination, it is important to determine the appropriate timings for vaccination spots and establish rapport with local community leaders to promote vaccination sites. For provider recommendations, educational campaigns should be implemented among health care workers and it is crucial to establish consistent and factual process of provider recommendation with on-going monitoring, including reminder and recall and incentives. Overall, provider recommendations and on-site vaccination should be employed along with other interventions to increase vaccination rates globally. Our results systematically expand upon the findings of the previous reviews conducted by Brewer et al^5^ and Oh et al^624^ and provide additional evidence including quantitative assessment of strategies to increase vaccine uptake rates.

## Supplementary Material

1

## Figures and Tables

**Table 1:** Distribution of studies included in the systematic review by domain and income status of the countries

Domains	Total Studies	Studies from HIC	No. of HIC represented	Studies from LMICs	No. of LMICs represented
Educational Campaigns	315	273	33	42	20
Reminder and Recall	155	136	16	19	9
Incentives	102	80	12	22	14
Message Framing	98	94	20	5	3
On-site vaccination	71	57	14	14	11
Free vaccination	63	54	17	9	5
Provider Recommendation	55	51	11	4	3
Institutional Recommendation	49	43	10	6	4
Vaccine Champion	23	21	5	2	1
