# Supplementary material for: Behavioral interventions for vaccination uptake: A systematic review and meta-analysis
Source: Health Policy. Author manuscript; Available in PMC 2024 Feb 23. (PMC10885629; doi:10.1016/j.healthpol.2023.104894)
Supplement: 1 [file NIHMS1946635-supplement-1.pdf]

Supplement

APPENDIX A

Approaches to identify final search strategy

Vaccination concept	exp vaccines/ or exp immunization/ or (vaccin* or immunis* or immuniz* or inoculat*).mp.			
Intervention study concept	(intervention or interventions or treatment or treatments or group or groups or trial or trials or program or programs or programme or programmes).mp. or (evaluat* or experiment*).ti,ab,kw.			
Date limit	1990-current			
Behavioral approaches concept	Operationalization I	Operationalization II	Operationalization III	Operationalization IV
Description	An early approach, using many potentially relevant search terms	Selected terms (based on their performance retrieving records in the validation set)	Fewer terms	Ultra-streamlined approach
Operationalizations of the behavioral approach concept	exp behavior/ or behav*.mp. or (incentiv* or presumptive communication or interpersonal communication or decision analysis or default).mp. or motivation/ or psychology.fs. or communication.mp. or ((risk or presumptive or interpersonal) adj1 communication).mp. or communication/ or exp communication/ or health	exp behavior/ or behav*.mp. or incentiv*.mp. or motivat*.mp. or motivation/ or psychology.fs. or communication.mp. or exp communication/ or (intent or intention*).mp. or (attitude or attitudes).mp. or exp attitude/ or (risk or risks).mp. or decision making.mp. or exp decision making/ or	exp behavior/ or behav*.mp. or incentiv*.mp. or psychology.fs. or	exp behavior/ or behav*.mp.

	communication/ or health literacy/ or (intent or intention*).mp. or intention/ or (persuad* or persuasi*).mp. or persuasive communication/ or narrative*.mp. or (message framing or messaging or health message*).mp. or affect.mp. or affect/ or social process*.mp. or (risk perception or (risk* adj2 (perception* or perceiv*))).mp. or (default or defaults).mp. or decision-making.mp. or (presumptive or participatory).mp. or (attitude or attitudes).mp. or exp attitude/ or attitude/ or attitude of health personnel/ or exp attitude to health/ or patient acceptance of health care/ or exp	(refus* or hesita* or delay* or activism or activist* or accept*).mp.		

	patient acceptance of health care/ or<br>(risk or risks).mp. or<br>((risk or risks) adj2 assess*).mp. or<br>exp risk/ or<br>risk/ or<br>risk assessment/ or<br>social network analys*.mp. or<br>(pro-vaccin* or anti-vaccin* or antivaccin*).mp. or<br>(pro-vax* or anti-vax* or antivax*).mp. or<br>anti-vaccination movement/ or<br>decision making.mp. or<br>exp decision making/ or<br>exp choice behavior/ or<br>decision making/ or<br>social norms/ or<br>social norm*.mp. or<br>exp reward/ or<br>motivation/ or<br>reward*.mp. or<br>cognition/ or<br>cognition.mp. or<br>exp cognition/ or<br>(intervention or interventions or			

	evaluation*).mp.<br>or<br>program evaluation/ or<br>((behavioral or behavioural) adj1 (economics or insight*)).mp. or<br>((behavior* or behaviour*) adj1 change theory).mp.<br>or<br>(behav* change adj1 (theor* or model*)).mp.			
Records retrieved by the behavioral concept*	7931390	6486942	3145763	26611721
Screening workload – union of vaccination, intervention study, and behavioral concepts, with date limit*	84363	59983	16112	14125
Performance retrieving validation articles available in Medline**	34/34<br>100%	31/33<br>91%	28/34<br>82%	19/34<br>56%
Comments	Sensitive, but not feasible	Sensitive, but not feasible	Feasible and relatively sensitive	Feasible but not sensitive

* The table presents information from testing on January 10, 2020. The same searches rerun today would retrieve additional records.

** As of January 2020, our validation article set included 34 articles available in Medline. As the testing process continued, we added additional articles to the validation set.

APPENDIX B

Final search Results

**Ovid MEDLINE(R) ALL <1946 to March 11, 2020>**		
Search history sorted by search number ascending		
**#**	**Searches**	**Results**
1	[concept 1 -- vaccines]	0
2	exp vaccines/	225103
3	exp immunization/	172624
4	(vaccin* or immunis* or immuniz* or inoculat*).mp.	569359
5	or/2-4	587159
6	[concept 2 -- intervention studies]	0
7	(intervention or interventions or treatment or treatments or group or groups or trial or trials or program or programs or programme or programmes).mp.	9225213
8	(evaluat* or experiment*).ti,ab,kw.	5218032
9	or/7-8	12097558
10	[concept 3 -- behavioral approaches]	0
11	exp behavior/	1772286
12	behav*.mp.	1675707
13	incentiv*.mp.	35679
14	psychology.fs.	1038196
15	motivat*.mp.	167355
16	motivation/	65829
17	or/11-16	3250486
18	5 and 9 and 17	18089
19	limit 18 to yr="1990 -Current"	16917
20	19 not (animals not humans).sh.	14753

APPENDIX C

The Grading of Recommendations Assessment, Development and Evaluation (GRADE) approach

GRADE approach assesses the quality of evidence and strength of recommendation in healthcare settings and is widely used during systematic review and clinical guidelines development processes.

The approach has 2 components:

1) Quality of evidence

Quality of evidence is rated as follows:

Grade	Description
High	There is a lot of confidence that the true effect lies close to that of the estimated effect.
Moderate	There is moderate confidence in the estimated effect: The true effect is likely to be close to the estimated effect, but there is a possibility that it is substantially different.
Low	There is limited effect in the estimated effect: The true effect might be substantially different from the estimated effect.
Very Low	There is very little confidence in the estimated effect: The true effect is likely to be substantially different from the estimated effect.

GRADE judgments refer not to individual studies but to a body of evidence. The quality of evidence for each patient important outcome is assessed based on study design and strengths and limitations present across the body of evidence. Randomized trials provide, in general, stronger evidence than observational studies. Rigorous observational studies provide stronger evidence than uncontrolled case series. In the GRADE approach, randomized trials without important limitations constitute high quality evidence. Observational studies without special strengths or important limitations constitute low quality evidence.

Limitations to consider:

a) Risk of bias (study limitations) including lack of allocation concealment; lack of blinding, particularly if outcomes are subjective and their assessment highly susceptible to bias; a large loss to follow-up; failure to adhere to an intention to treat analysis; stopping early for benefit; or selective reporting of outcomes
b) Inconsistent results across studies
c) Indirectness of evidence
d) Imprecision of estimate
e) Publication bias

Strengths to consider:

a) Size of the effect estimate. Larger the magnitude of effect, stronger the evidence
b) Identification of bias working against the treatment effect
c) Presence of dose-response gradient

GRADE starts with the study design to rate the quality of evidence and then rates it downwards or upwards depending on the limitations and strengths as shown in the table below:

A summary of GRADE's approach to rating quality of evidence

Study design	Initial quality of a body of evidence	Lower if	Higher if	Quality of a body of evidence
Randomized trials	High	Risk of Bias<br>−1 Serious<br>−2 Very serious	Large effect<br>+1 Large<br>+2 Very large	High (four plus: ⊕⊕⊕⊕)
		Inconsistency<br>−1 Serious<br>−2 Very serious	Dose response<br>+1 Evidence of a gradient	Moderate (three plus: ⊕⊕⊕○)
Observational studies	Low	Indirectness<br>−1 Serious<br>−2 Very serious	All plausible residual confounding<br>+1 Would reduce a demonstrated effect<br>+1 Would suggest a spurious effect if no effect was observed	Low (two plus: ⊕⊕○○)
		Imprecision<br>−1 Serious<br>−2 Very serious		Very low (one plus: ⊕○○○)
		Publication bias<br>−1 Likely<br>−2 Very likely		

2) Strength of recommendation

GRADE rates strength of recommendation either as strong or weak. Strong recommendation is made when desirable effects certainly outweigh undesirable effects (or vice versa) while a weak recommendation is made when there is more uncertainty present.

Things to consider while rating strength of recommendation:

a) Quality of evidence
b) Uncertainty about the balance between desirable and undesirable effects
c) Uncertainty or variability in values and preferences
d) Uncertainty about whether the intervention represents a wise use of resources

References

1. Guyatt GH, Oxman AD, Vist GE, Kunz R, Falck-Ytter Y, Alonso-Coello P, Schünemann HJ. GRADE: an emerging consensus on rating quality of evidence and strength of recommendations. BMJ. 2008 Apr 24;336(7650):924-6.
2. Guyatt GH, Oxman AD, Kunz R, Vist GE, Falck-Ytter Y, Schünemann HJ. What is "quality of evidence" and why is it important to clinicians?. BMJ. 2008 May 1;336(7651):995-8.
3. Balshem H, Helfand M, Schünemann HJ, Oxman AD, Kunz R, Brozek J, Vist GE, Falck-Ytter Y, Meerpohl J, Norris S, Guyatt GH. GRADE guidelines: 3. Rating the quality of evidence. Journal of Clinical Epidemiology. 2011 Apr 1;64(4):401-6.
4. Guyatt G, Oxman AD, Sultan S, Brozek J, Glasziou P, Alonso-Coello P, Atkins D, Kunz R, Montori V, Jaeschke R, Rind D. GRADE guidelines: 11. Making an overall rating of confidence in effect estimates for a single outcome and for all outcomes. Journal of Clinical Epidemiology. 2013 Feb 1;66(2):151-7.
5. Andrews JC, Schünemann HJ, Oxman AD, Pottie K, Meerpohl JJ, Coello PA, Rind D, Montori VM, Brito JP, Norris S, Elbarbary M. GRADE guidelines: 15. Going from evidence to recommendation—determinants of a recommendation's direction and strength. Journal of Clinical Epidemiology. 2013 Jul 1;66(7):726-35.

Supplement Figure 1. Geographical Distribution of Countries includes in the Systematic Review

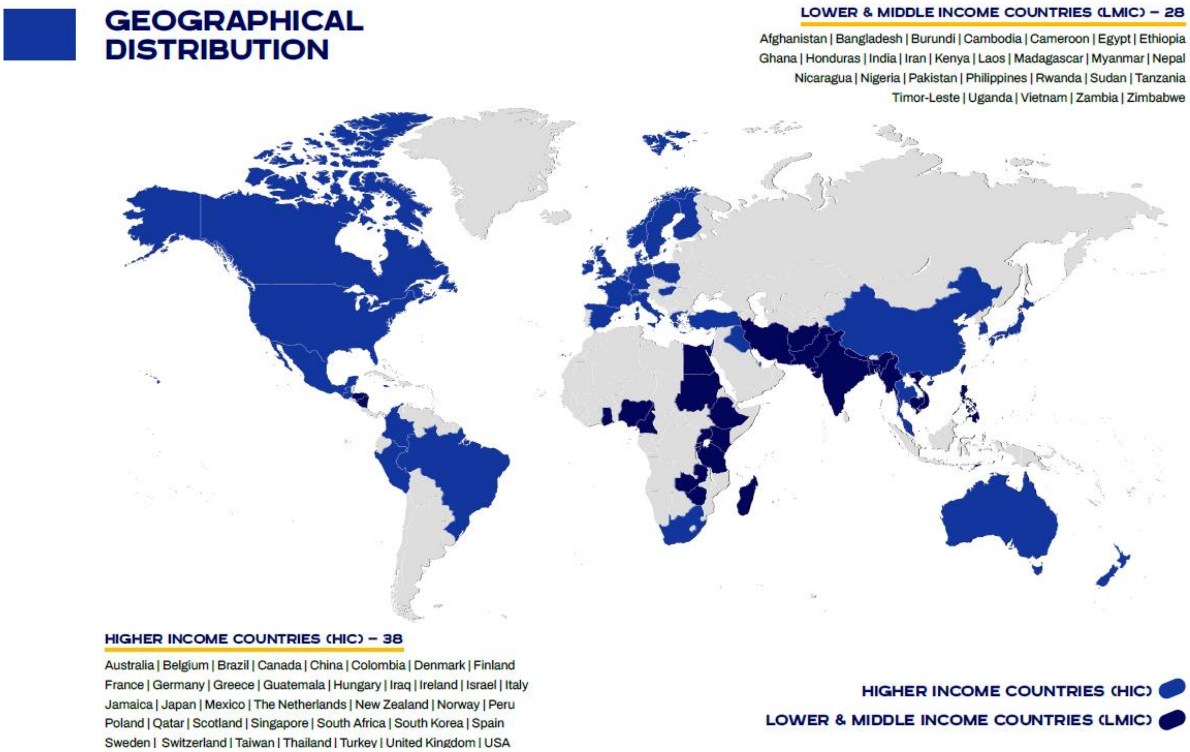

Supplement Figure 2. Forrest Plot showing ORs and respective 95% CIs for all RCTs from LMICs included in the meta-analysis by domains

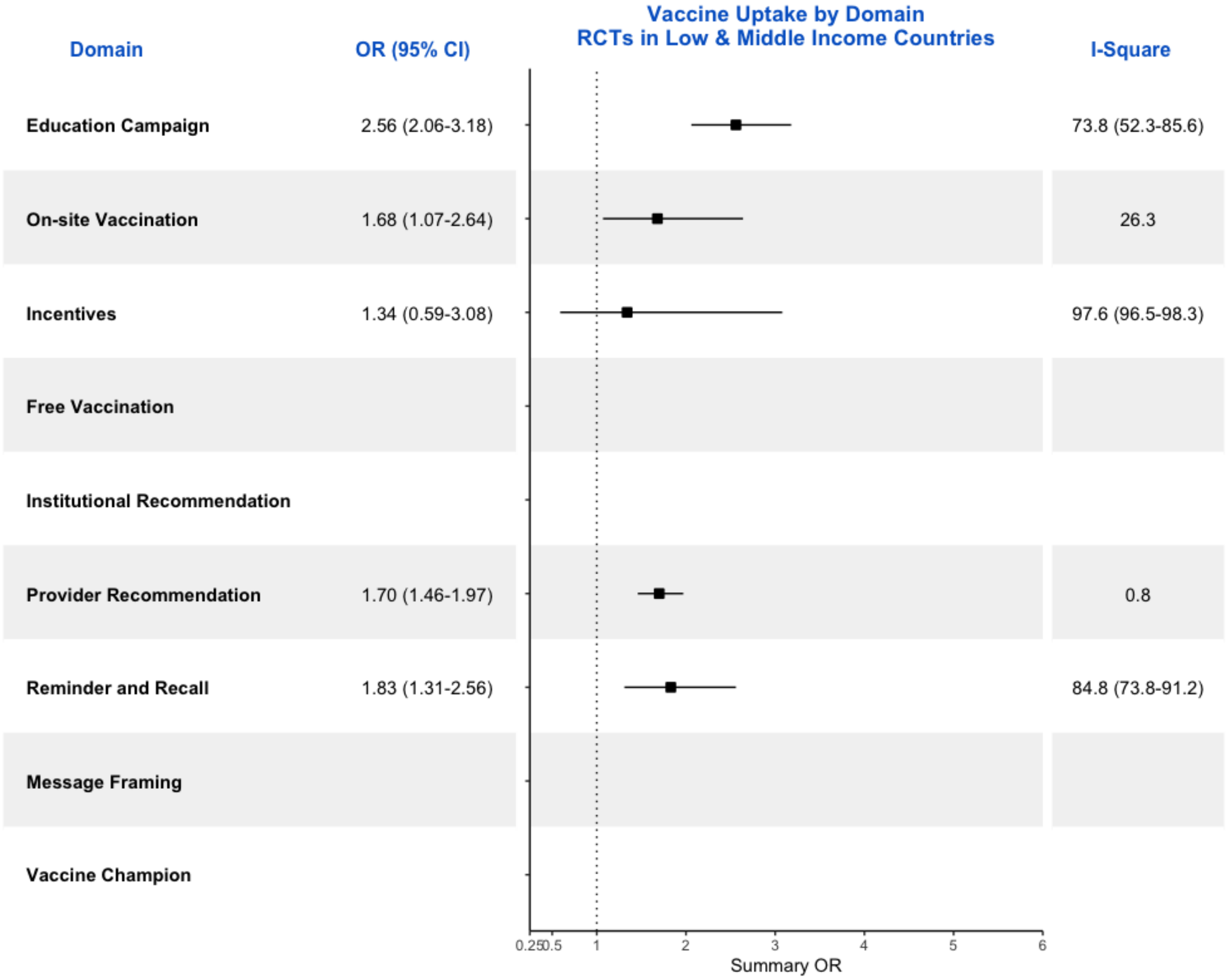

Supplement Figure 3. Funnel Plots showing distribution of ORs and SEs of all studies by domains

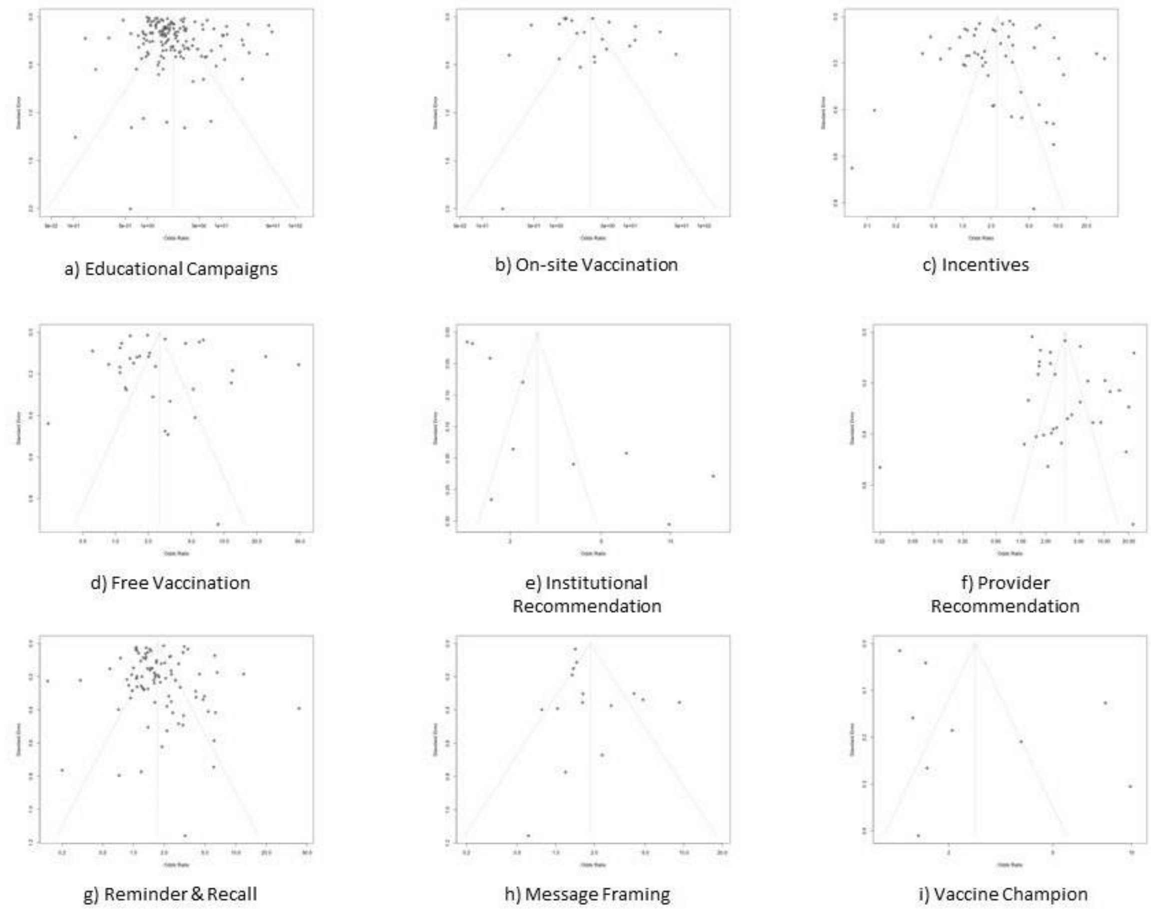

Supplemental Table 1. Approaches to identify final search strategy.

Vaccination concept	exp vaccines/ or exp immunization/ or (vaccin* or immunis* or immuniz* or inoculat*).mp.			
Intervention study concept	(intervention or interventions or treatment or treatments or group or groups or trial or trials or program or programs or programme or programmes).mp. or (evaluat* or experiment*).ti,ab,kw.			
Date limit	1990-current			
Behavioral approaches concept	Operationalization I	Operationalization II	Operationalization III	Operationalization IV
Description	An early approach, using many potentially relevant search terms	Selected terms (based on their performance retrieving records in the validation set)	Fewer terms	Ultra-streamlined approach
Records retrieved by the behavioral concept*	7931390	6486942	3145763	26611721
Screening workload *	84363	59983	16112	14125
Performance retrieving validation articles available in Medline**	34/34<br>100%	31/34<br>91%	28/34<br>82%	19/34<br>56%

* The table presents information from testing on January 10, 2020. The same searches rerun today would retrieve additional records.

** As of January 2020, our validation article set included 34 articles available in Medline. As the testing process continued, we added additional articles to the validation set.

Supplement Table 2. ORs and respective 95% CIs for all studies included in the meta-analysis by domains after removing the outlier studies

	N (meta)	OR (95% CI)	I-square (%)
**Education Campaign**	77	2.1 (2.0-2.2)	40.3
**On-site Vaccination**	10	2.7 (2.2-3.5)	59.4
**Incentives**	20	2.3 (2.1-2.6)	72.5
**Free Vaccination**	15	2.3 (1.9-2.7)	89.9
**Institutional Recommendation**	4	2.4 (1.8-3.1)	60.0
**Provider Recommendation**	18	3.1 (2.6-3.8)	77.6
**Reminder and Recall**	52	1.6 (1.5-1.7)	38.6
**Message Framing**	13	1.5 (1.3-1.8)	39.2
**Vaccine Champion**	5	2.0 (1.4-2.9)	71.7

Supplement Table 3. Results of Egger's Regression for all studies and RCTs included in the meta-analysis by domains

	All Studies		RCTs	
	Intercept (95% CI)	p-value	Intercept (95% CI)	p-value
**Education Campaign**	4.4 (2.6-6.2)	<0.01	0.9 (-0.7-2.5)	0.25
**On-site Vaccination**	3.7 (-2.4-9.9)	0.25	0.7 (-5.0-6.5)	0.81
**Incentives**	-1.6 (-5.1-1.8)	0.35	-2.8 (-8.1-2.4)	0.30
**Free Vaccination**	2.6 (-2.1-7.2)	0.29	-3.8 (-17.1-9.6)	0.60
**Institutional Recommendation**	6.4 (4.0-8.9)	<0.01	3.0 (-0.4-5.7)	0.15
**Provider Recommendation**	3.9 (0.5-7.3)	0.03	1.0 (-1.0-3.1)	0.37
**Reminder and Recall**	-0.6 (-2.3-1.2)	0.52	1.6 (0.02-3.3)	0.05
**Message Framing**	1.0 (-0.3-2.2)	0.15	0.5 (-1.1-2.1)	0.56
**Vaccine Champion**	4.7 (0.9-8.6)	0.04	2.3 (0.1-4.6)	0.14

Supplement Table 4. Adjusted ORs and respective 95%CIs using Trim and Fill method for outlier studies removed for all studies included in the meta-analysis by domains

	N	OR (95% CI)	I-square (%)
**Education Campaign**	77 (0 added studies)	2.1 (2.0-2.2)	40.3
**Institutional Recommendation**	4 (0 added studies)	2.4 (1.8-3.1)	60
**Provider Recommendation**	20 (2 added studies)	3.4 (2.8-4.1)	77.7
**Vaccine Champion**	5 (0 added studies)	2.0 (1.4-2.9)	71.7
